# (*E*)-2,2′-[3-(4-Chloro­phen­yl)prop-2-ene-1,1-di­yl]bis­(3-hy­droxy-5,5-di­methyl­cyclo­hex-2-en-1-one)

**DOI:** 10.1107/S1600536813020357

**Published:** 2013-07-27

**Authors:** Joo Hwan Cha, Jae Kyun Lee, Sun-Joon Min, Yong Seo Cho, Junghwan Park

**Affiliations:** a Advanced Analysis Center, Korea Institute of Science & Technology, Hwarangro 14-gil, Seongbuk-gu, Seoul 136-791, Republic of Korea; b Center for Neuro-Medicine, Korea Institute of Science & Technology, Hwarangro 14-gil, Seongbuk-gu, Seoul 136-791, Republic of Korea; c Corporated R&D Center, Duksan Hi-Metal Co. Ltd., Cheonan-si 331-821, Republic of Korea

## Abstract

The title compound, C_25_H_29_ClO_4_, adopts a *trans* conformation about the C=C double bond and the di­methyl­cyclo­hexenone rings both show an envelope conformation with the dimethyl-substituted C atom as the flap. In the mol­ecule, the hy­droxy and carbonyl groups form two intra­molecular O—H⋯O hydrogen bonds typical for xanthene derivatives. In the crystal, weak C—H⋯O hydrogen bonds link the mol­ecules into chains running parallel to the *a*-axis direction.

## Related literature

For the crystal structures of xanthene derivatives studied recently our group, see: Cha *et al.* (2011 ▶, 2012 ▶, 2013 ▶).
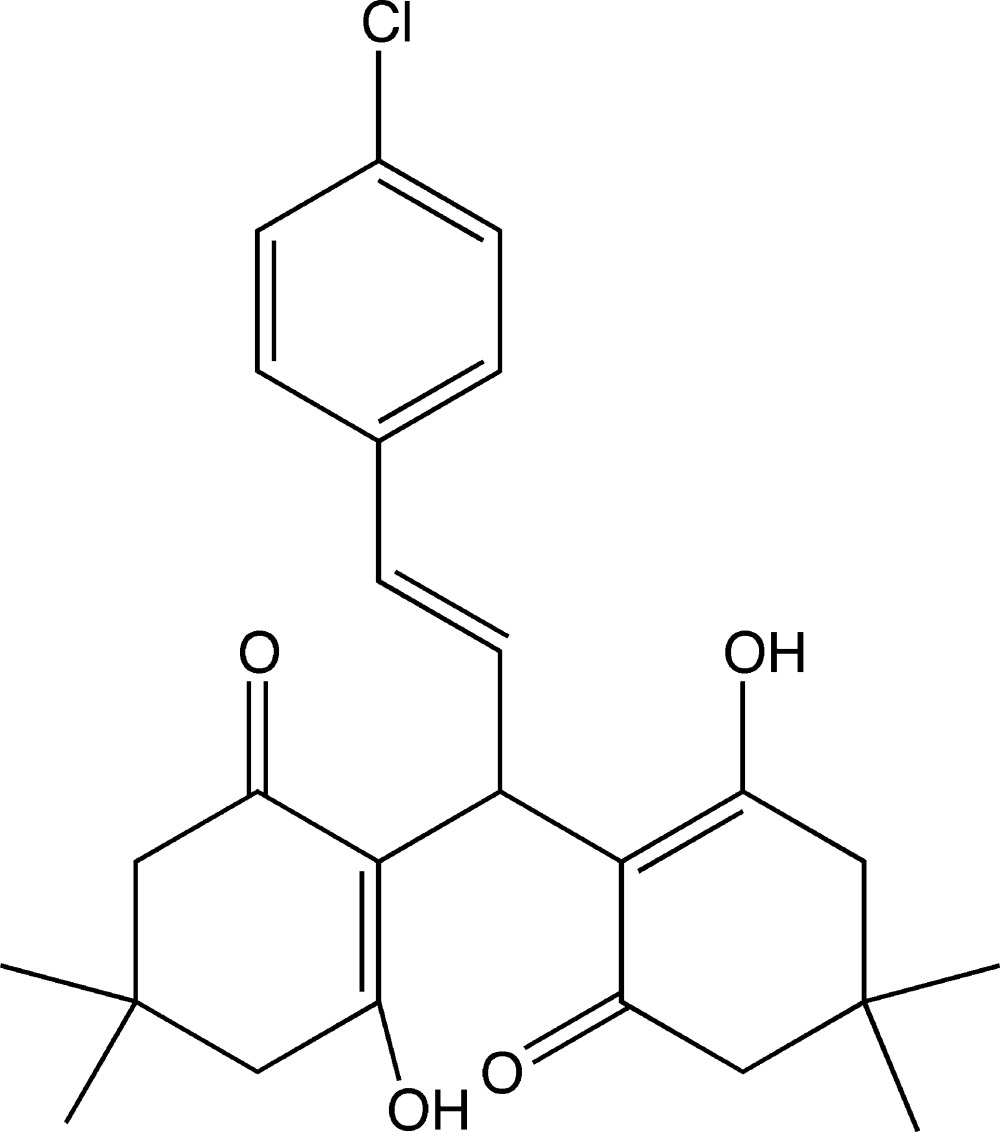


## Experimental

### 

#### Crystal data

C_25_H_29_ClO_4_
*M* *_r_* = 428.95
Monoclinic, 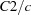

*a* = 25.8781 (16) Å
*b* = 9.7820 (6) Å
*c* = 20.9904 (11) Å
β = 121.2919 (15)°
*V* = 4540.5 (5) Å^3^
*Z* = 8
Mo *K*α radiation
μ = 0.20 mm^−1^
*T* = 296 K
0.20 × 0.20 × 0.20 mm

#### Data collection

Rigaku R-AXIS RAPID diffractometer
Absorption correction: multi-scan (*ABSCOR*; Rigaku, 1995 ▶) *T*_min_ = 0.692, *T*_max_ = 0.962
21331 measured reflections
5175 independent reflections
3926 reflections with *F* ^2^ > 2σ(*F* ^2^)
*R*_int_ = 0.028

#### Refinement

*R*[*F* ^2^ > 2σ(*F* ^2^)] = 0.045
*wR*(*F* ^2^) = 0.135
*S* = 1.08
5175 reflections
281 parameters
H atoms treated by a mixture of independent and constrained refinement
Δρ_max_ = 0.31 e Å^−3^
Δρ_min_ = −0.28 e Å^−3^

### 

Data collection: *RAPID-AUTO* (Rigaku, 2006 ▶); cell refinement: *RAPID-AUTO*; data reduction: *RAPID-AUTO*; program(s) used to solve structure: *Il Milione* (Burla *et al.*, 2007 ▶); program(s) used to refine structure: *SHELXL97* (Sheldrick, 2008 ▶); molecular graphics: *CrystalStructure* (Rigaku, 2010 ▶); software used to prepare material for publication: *CrystalStructure*.

## Supplementary Material

Crystal structure: contains datablock(s) General, I. DOI: 10.1107/S1600536813020357/ff2113sup1.cif

Structure factors: contains datablock(s) I. DOI: 10.1107/S1600536813020357/ff2113Isup2.hkl

Additional supplementary materials:  crystallographic information; 3D view; checkCIF report

## Figures and Tables

**Table 1:** Hydrogen-bond geometry (Å, °)

*D*—H⋯*A*	*D*—H	H⋯*A*	*D*⋯*A*	*D*—H⋯*A*
O2—H2⋯O1	0.82	1.78	2.596 (2)	173
O4—H4⋯O3	0.82	1.87	2.669 (2)	166
C18—H18⋯O2^i^	0.93	2.51	3.418 (3)	165
C21—H21⋯O3^ii^	0.93	2.61	3.397 (3)	143

Symmetry codes: (i) 
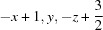
; (ii) 
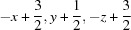
.

**Fig. 1.**
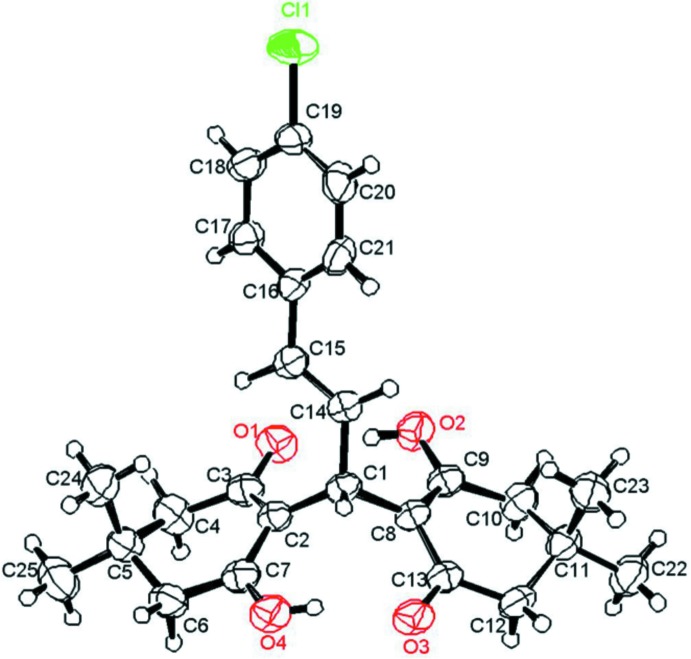
The molecular structure of (I) showing the atomic numbering and 50% probability displacement ellipsoid.

## supplementary crystallographic information

### Comment

As part of our ongoing study of the substituent effect on the solid state structures of dimethyl cyclohexenone ring derivatives (Cha *et al.*, 2011, 2012, 2013) we present here the crystal structure of the title compound, C_25_H_29_ClO_4_. In the title compound (Fig. 1), the bond lengths and angles are normal and correspond to those observed in related structures. Both dimethyl cyclohexenone rings (C2—C7), (C8—C13) show an envelope conformation. The hydroxy and carbonyl O atoms face each other and are oriented to allow for the formation of two intramolecular O—H···O hydrogen bonds. In the crystal, weak intermolecular C—H···O hydrogen bonds (Table 1) link molecules into chains running parallel to the *a*-axis.

### Experimental

To solution of 5,5-Dimethyl-1,3-cyclohexanedione (4.61 mmol), 4-chlorocinnamaldehyde(1.84 mmol) and 4 Å molecular sieve (M.S.) was added catalytic amounts of *L*-proline under nitrogen atmosphere. The anhydrous ethyl acetate (2 ml) was added to a reaction mixture and the solution was stirred at room temperature for 6 h. The progress of reaction was monitored by TLC. After completion of reaction, the reaction mixture was filtered through aa pad of celite to remove M.S. and evaporation of the solvent afforded a mixture. The mixture was purified by flash column chromatography (E.A.: Hexane = 1:3) to afford the title compound as a colorless solid in yield 91%. Recrystallization from ethanol gave crystals suitable for X-ray analysis.

### Refinement

All hydrogen atoms were positioned geometrically and refined using a riding model with C—H = 0.82–1.03 Å and *U*iso(H) = 1.2 or 1.5 *U*eq(C).

### Crystal data

**Table:**

C_25_H_29_ClO_4_	*F*(000) = 1824.00
*M**_r_* = 428.95	*D*_x_ = 1.255 Mg m^−^^3^
Monoclinic, *C*2/*c*	Mo *K*α radiation, λ = 0.71075 Å
Hall symbol: -C 2yc	Cell parameters from 15174 reflections
*a* = 25.8781 (16) Å	θ = 3.2–27.5°
*b* = 9.7820 (6) Å	µ = 0.20 mm^−^^1^
*c* = 20.9904 (11) Å	*T* = 296 K
β = 121.2919 (15)°	Block, colourless
*V* = 4540.5 (5) Å^3^	0.20 × 0.20 × 0.20 mm
*Z* = 8	

### Data collection

**Table:**

Rigaku R-AXIS RAPID diffractometer	3926 reflections with *F*^2^ > 2σ(*F*^2^)
Detector resolution: 10.000 pixels mm^-1^	*R*_int_ = 0.028
ω scans	θ_max_ = 27.5°
Absorption correction: multi-scan (*ABSCOR*; Rigaku, 1995)	*h* = −33→33
*T*_min_ = 0.692, *T*_max_ = 0.962	*k* = −12→12
21331 measured reflections	*l* = −27→24
5175 independent reflections	

### Refinement

**Table:**

Refinement on *F*^2^	Secondary atom site location: difference Fourier map
*R*[*F*^2^ > 2σ(*F*^2^)] = 0.045	Hydrogen site location: inferred from neighbouring sites
*wR*(*F*^2^) = 0.135	H atoms treated by a mixture of independent and constrained refinement
*S* = 1.08	*w* = 1/[σ^2^(*F*_o_^2^) + (0.0716*P*)^2^ + 1.3045*P*] where *P* = (*F*_o_^2^ + 2*F*_c_^2^)/3
5175 reflections	(Δ/σ)_max_ < 0.001
281 parameters	Δρ_max_ = 0.31 e Å^−^^3^
0 restraints	Δρ_min_ = −0.28 e Å^−^^3^
Primary atom site location: structure-invariant direct methods	

### Special details

**Table:**

Geometry. All e.s.d.'s (except the e.s.d. in the dihedral angle between two l.s. planes) are estimated using the full covariance matrix. The cell e.s.d.'s are taken into account individually in the estimation of e.s.d.'s in distances, angles and torsion angles; correlations between e.s.d.'s in cell parameters are only used when they are defined by crystal symmetry. An approximate (isotropic) treatment of cell e.s.d.'s is used for estimating e.s.d.'s involving l.s. planes.
Refinement. Refinement was performed using all reflections. The weighted *R*-factor (*wR*) and goodness of fit (*S*) are based on *F*^2^. *R*-factor (gt) are based on *F*. The threshold expression of *F*^2^ > 2.0 σ(*F*^2^) is used only for calculating *R*-factor (gt).

### Fractional atomic coordinates and isotropic or equivalent isotropic displacement parameters (Å^2^)

**Table:**

	*x*	*y*	*z*	*U*_iso_*/*U*_eq_	
Cl1	0.60735 (3)	0.86965 (6)	1.00738 (3)	0.0801 (2)	
O1	0.53057 (5)	0.38582 (12)	0.63842 (7)	0.0500 (3)	
O2	0.56169 (5)	0.60824 (13)	0.59936 (7)	0.0537 (3)	
O3	0.73761 (5)	0.38291 (13)	0.64466 (7)	0.0527 (3)	
O4	0.71474 (5)	0.17267 (13)	0.70741 (7)	0.0538 (3)	
C1	0.66126 (6)	0.43105 (16)	0.70337 (8)	0.0374 (3)	
C2	0.62898 (6)	0.29467 (15)	0.68798 (8)	0.0374 (3)	
C3	0.56690 (7)	0.28289 (16)	0.66028 (8)	0.0414 (4)	
C4	0.53791 (8)	0.14617 (18)	0.65355 (11)	0.0536 (5)	
C5	0.58134 (8)	0.03632 (17)	0.70514 (9)	0.0489 (4)	
C6	0.63474 (8)	0.03533 (17)	0.69324 (10)	0.0513 (4)	
C7	0.66125 (7)	0.17326 (17)	0.69678 (8)	0.0427 (4)	
C8	0.65497 (6)	0.50368 (16)	0.63540 (8)	0.0372 (3)	
C9	0.60755 (7)	0.59492 (16)	0.59183 (8)	0.0416 (4)	
C10	0.60661 (7)	0.68081 (18)	0.53225 (9)	0.0474 (4)	
C11	0.66824 (8)	0.69941 (17)	0.53963 (8)	0.0453 (4)	
C12	0.69780 (8)	0.55825 (18)	0.55360 (9)	0.0474 (4)	
C13	0.69722 (7)	0.47803 (16)	0.61431 (8)	0.0407 (4)	
C14	0.65213 (7)	0.52792 (17)	0.75347 (8)	0.0403 (4)	
C15	0.62794 (7)	0.49923 (17)	0.79349 (9)	0.0441 (4)	
C16	0.62435 (7)	0.59311 (15)	0.84652 (8)	0.0399 (4)	
C17	0.57709 (7)	0.57897 (17)	0.85930 (9)	0.0447 (4)	
C18	0.57174 (8)	0.66411 (18)	0.90809 (10)	0.0489 (4)	
C19	0.61431 (8)	0.76360 (16)	0.94535 (8)	0.0460 (4)	
C20	0.66228 (8)	0.78006 (18)	0.93498 (10)	0.0539 (5)	
C21	0.66711 (8)	0.69360 (19)	0.88557 (9)	0.0505 (4)	
C22	0.65859 (10)	0.7597 (3)	0.46664 (10)	0.0627 (5)	
C23	0.70827 (9)	0.7961 (2)	0.60417 (10)	0.0576 (5)	
C24	0.60344 (11)	0.0684 (3)	0.78706 (11)	0.0652 (5)	
C25	0.55002 (11)	−0.1028 (2)	0.68548 (14)	0.0720 (6)	
H1	0.7042	0.4070	0.7331	0.0449*	
H2	0.5548	0.5353	0.6128	0.0645*	
H4A	0.5195	0.1148	0.6024	0.0643*	
H4B	0.5059	0.1576	0.6642	0.0643*	
H4	0.7209	0.2451	0.6928	0.0646*	
H6A	0.6662	−0.0229	0.7308	0.0616*	
H6B	0.6216	−0.0049	0.6449	0.0616*	
H10A	0.5907	0.7703	0.5328	0.0569*	
H10B	0.5791	0.6394	0.4842	0.0569*	
H12A	0.6770	0.5059	0.5077	0.0569*	
H12B	0.7394	0.5697	0.5666	0.0569*	
H14	0.6705 (9)	0.616 (2)	0.7603 (11)	0.057 (6)*	
H15	0.6082 (9)	0.405 (3)	0.7873 (11)	0.060 (6)*	
H17	0.5484	0.5108	0.8345	0.0537*	
H18	0.5395	0.6540	0.9156	0.0587*	
H20	0.6910	0.8478	0.9606	0.0647*	
H21	0.6996	0.7035	0.8787	0.0606*	
H22A	0.6970	0.7701	0.4704	0.0753*	
H22B	0.6393	0.8473	0.4578	0.0753*	
H22C	0.6335	0.6994	0.4261	0.0753*	
H23A	0.7128	0.7608	0.6495	0.0691*	
H23B	0.6898	0.8848	0.5941	0.0691*	
H23C	0.7473	0.8032	0.6093	0.0691*	
H24A	0.5694	0.0726	0.7936	0.0783*	
H24B	0.6241	0.1547	0.8004	0.0783*	
H24C	0.6306	−0.0021	0.8183	0.0783*	
H25A	0.5372	−0.1253	0.6349	0.0864*	
H25B	0.5154	−0.0994	0.6910	0.0864*	
H25C	0.5777	−0.1711	0.7183	0.0864*	

### Atomic displacement parameters (Å^2^)

**Table:**

	*U*^11^	*U*^22^	*U*^33^	*U*^12^	*U*^13^	*U*^23^
Cl1	0.1143 (5)	0.0719 (4)	0.0670 (4)	−0.0022 (3)	0.0562 (4)	−0.0239 (3)
O1	0.0344 (6)	0.0510 (7)	0.0608 (7)	0.0095 (5)	0.0220 (6)	0.0033 (6)
O2	0.0445 (7)	0.0595 (8)	0.0684 (8)	0.0160 (6)	0.0371 (6)	0.0161 (6)
O3	0.0411 (7)	0.0610 (8)	0.0635 (7)	0.0144 (6)	0.0323 (6)	0.0061 (6)
O4	0.0441 (7)	0.0592 (8)	0.0618 (7)	0.0185 (6)	0.0301 (6)	0.0112 (6)
C1	0.0318 (7)	0.0474 (8)	0.0338 (7)	0.0066 (6)	0.0175 (6)	0.0018 (6)
C2	0.0360 (8)	0.0434 (8)	0.0335 (7)	0.0063 (6)	0.0185 (6)	0.0021 (6)
C3	0.0376 (8)	0.0474 (8)	0.0382 (7)	0.0049 (7)	0.0191 (7)	−0.0008 (7)
C4	0.0434 (10)	0.0510 (10)	0.0597 (10)	−0.0014 (8)	0.0221 (8)	−0.0032 (8)
C5	0.0546 (10)	0.0429 (9)	0.0504 (9)	0.0005 (7)	0.0281 (8)	−0.0027 (7)
C6	0.0593 (11)	0.0446 (9)	0.0505 (9)	0.0115 (8)	0.0289 (9)	0.0012 (7)
C7	0.0418 (8)	0.0515 (9)	0.0338 (7)	0.0101 (7)	0.0188 (7)	0.0024 (7)
C8	0.0328 (7)	0.0458 (8)	0.0355 (7)	0.0031 (6)	0.0195 (6)	−0.0009 (6)
C9	0.0367 (8)	0.0497 (9)	0.0390 (8)	0.0027 (7)	0.0202 (7)	−0.0007 (7)
C10	0.0432 (9)	0.0570 (10)	0.0393 (8)	0.0050 (8)	0.0195 (7)	0.0072 (7)
C11	0.0491 (9)	0.0522 (9)	0.0382 (8)	−0.0021 (8)	0.0253 (7)	−0.0001 (7)
C12	0.0500 (10)	0.0572 (10)	0.0471 (9)	−0.0022 (8)	0.0337 (8)	−0.0047 (8)
C13	0.0360 (8)	0.0484 (9)	0.0412 (8)	−0.0001 (7)	0.0225 (7)	−0.0048 (7)
C14	0.0410 (8)	0.0444 (8)	0.0379 (8)	0.0011 (7)	0.0221 (7)	−0.0011 (7)
C15	0.0481 (9)	0.0453 (9)	0.0453 (8)	−0.0020 (7)	0.0288 (8)	−0.0028 (7)
C16	0.0443 (9)	0.0419 (8)	0.0371 (7)	0.0007 (7)	0.0237 (7)	0.0024 (6)
C17	0.0418 (9)	0.0490 (9)	0.0470 (8)	−0.0074 (7)	0.0255 (7)	−0.0081 (7)
C18	0.0475 (10)	0.0537 (9)	0.0554 (10)	−0.0005 (8)	0.0336 (8)	−0.0053 (8)
C19	0.0597 (10)	0.0433 (8)	0.0389 (8)	0.0020 (8)	0.0284 (8)	−0.0032 (7)
C20	0.0606 (11)	0.0494 (9)	0.0495 (9)	−0.0182 (8)	0.0270 (9)	−0.0071 (8)
C21	0.0517 (10)	0.0588 (10)	0.0527 (9)	−0.0102 (8)	0.0353 (8)	0.0005 (8)
C22	0.0753 (13)	0.0712 (13)	0.0517 (10)	−0.0018 (10)	0.0400 (10)	0.0076 (9)
C23	0.0613 (11)	0.0572 (11)	0.0523 (10)	−0.0086 (9)	0.0281 (9)	−0.0064 (8)
C24	0.0855 (15)	0.0636 (12)	0.0579 (11)	−0.0058 (11)	0.0452 (11)	0.0004 (9)
C25	0.0824 (16)	0.0480 (10)	0.0873 (15)	−0.0073 (10)	0.0452 (13)	−0.0059 (10)

### Geometric parameters (Å, º)

**Table:**

Cl1—C19	1.746 (2)	C20—C21	1.393 (3)
O1—C3	1.288 (2)	O2—H2	0.820
O2—C9	1.283 (3)	O4—H4	0.820
O3—C13	1.2932 (19)	C1—H1	0.980
O4—C7	1.283 (3)	C4—H4A	0.970
C1—C2	1.517 (3)	C4—H4B	0.970
C1—C8	1.525 (3)	C6—H6A	0.970
C1—C14	1.522 (3)	C6—H6B	0.970
C2—C3	1.401 (3)	C10—H10A	0.970
C2—C7	1.408 (3)	C10—H10B	0.970
C3—C4	1.504 (3)	C12—H12A	0.970
C4—C5	1.525 (3)	C12—H12B	0.970
C5—C6	1.527 (4)	C14—H14	0.96 (2)
C5—C24	1.537 (3)	C15—H15	1.03 (3)
C5—C25	1.527 (3)	C17—H17	0.930
C6—C7	1.498 (3)	C18—H18	0.930
C8—C9	1.405 (2)	C20—H20	0.930
C8—C13	1.399 (3)	C21—H21	0.930
C9—C10	1.496 (3)	C22—H22A	0.960
C10—C11	1.531 (3)	C22—H22B	0.960
C11—C12	1.531 (3)	C22—H22C	0.960
C11—C22	1.536 (3)	C23—H23A	0.960
C11—C23	1.531 (3)	C23—H23B	0.960
C12—C13	1.503 (3)	C23—H23C	0.960
C14—C15	1.312 (3)	C24—H24A	0.960
C15—C16	1.483 (3)	C24—H24B	0.960
C16—C17	1.388 (3)	C24—H24C	0.960
C16—C21	1.386 (3)	C25—H25A	0.960
C17—C18	1.382 (3)	C25—H25B	0.960
C18—C19	1.370 (3)	C25—H25C	0.960
C19—C20	1.377 (4)		
			
O1···O2	2.596 (2)	H22B···H23C	2.9787
O1···C1	2.9500 (19)	H22C···H23B	3.5386
O1···C7	3.600 (2)	H22C···H23C	3.5689
O1···C8	3.451 (3)	H24A···H25A	3.5586
O1···C9	3.337 (3)	H24A···H25B	2.5096
O1···C14	3.1254 (18)	H24A···H25C	2.9303
O1···C15	3.1072 (18)	H24B···H25B	3.5566
O2···C1	2.9268 (17)	H24B···H25C	3.5249
O2···C2	3.5406 (19)	H24C···H25A	3.5295
O2···C3	3.406 (2)	H24C···H25B	2.9424
O2···C13	3.589 (3)	H24C···H25C	2.4564
O2···C14	2.9486 (17)	Cl1···H6B^i^	3.0186
O3···O4	2.669 (2)	Cl1···H22B^xi^	3.2133
O3···C1	2.859 (3)	Cl1···H23B^xi^	3.0987
O3···C2	3.483 (3)	Cl1···H25A^viii^	3.3630
O3···C7	3.399 (3)	O1···H10B^ii^	2.6745
O4···C1	2.862 (2)	O1···H17^iii^	2.6842
O4···C3	3.584 (3)	O1···H18^iii^	3.0529
O4···C8	3.568 (2)	O2···H18^iii^	2.5113
O4···C13	3.468 (2)	O2···H25A^iv^	2.8705
C2···C5	2.915 (3)	O2···H25C^iv^	3.1597
C2···C9	3.442 (3)	O3···H6A^vii^	2.6688
C2···C13	3.401 (3)	O3···H14^v^	3.395 (18)
C2···C15	2.995 (3)	O3···H20^v^	3.5562
C2···C24	3.330 (4)	O3···H21^v^	2.6046
C3···C6	2.857 (3)	O3···H24C^vii^	3.2782
C3···C8	3.372 (3)	O4···H1^v^	3.1608
C3···C14	3.155 (2)	O4···H14^v^	2.74 (3)
C3···C15	3.194 (3)	O4···H21^v^	3.5252
C3···C24	3.125 (3)	O4···H23A^v^	2.7257
C4···C7	2.841 (3)	O4···H23B^ix^	3.5219
C7···C8	3.452 (3)	C1···H23A^v^	3.5318
C7···C24	3.135 (4)	C1···H23C^v^	3.5995
C8···C11	2.923 (3)	C3···H10B^ii^	3.4852
C8···C23	3.384 (3)	C4···H10B^ii^	3.5823
C9···C12	2.859 (4)	C4···H24A^iii^	3.5584
C9···C14	3.034 (3)	C6···H23B^ix^	3.4066
C9···C23	3.167 (3)	C7···H23A^v^	3.2924
C10···C13	2.864 (3)	C12···H22A^xii^	3.4517
C13···C23	3.142 (3)	C13···H6A^vii^	3.3361
C14···C21	3.058 (3)	C14···H4^vii^	3.5679
C16···C19	2.777 (3)	C14···H23C^v^	3.4805
C17···C20	2.760 (3)	C14···H25C^iv^	3.3828
C18···C21	2.759 (4)	C15···H22C^i^	3.3353
Cl1···C7^i^	3.4881 (18)	C15···H23C^v^	3.3798
O1···C10^ii^	3.5567 (17)	C15···H25C^iv^	3.5282
O1···C17^iii^	3.387 (3)	C16···H12A^i^	3.0750
O1···C18^iii^	3.562 (3)	C16···H22C^i^	3.2586
O2···C18^iii^	3.418 (3)	C16···H25C^iv^	3.2630
O2···C25^iv^	3.452 (3)	C17···H10B^i^	3.3620
O3···C21^v^	3.397 (3)	C17···H12A^i^	2.9514
O4···C14^v^	3.407 (3)	C17···H22C^i^	3.0627
O4···C23^v^	3.589 (3)	C18···H2^iii^	3.3192
C7···Cl1^vi^	3.4881 (18)	C18···H10B^i^	3.3305
C10···O1^ii^	3.5567 (17)	C18···H12A^i^	2.9391
C14···O4^vii^	3.407 (3)	C18···H25A^viii^	3.2196
C17···O1^iii^	3.387 (3)	C18···H25B^viii^	3.1455
C18···O1^iii^	3.562 (3)	C19···H12A^i^	3.0146
C18···O2^iii^	3.418 (3)	C19···H25A^viii^	3.5403
C18···C25^viii^	3.544 (3)	C19···H25B^viii^	3.3584
C21···O3^vii^	3.397 (3)	C20···H12A^i^	3.1142
C23···O4^vii^	3.589 (3)	C20···H20^xiii^	3.4842
C25···O2^ix^	3.452 (3)	C20···H24C^iv^	3.0203
C25···C18^x^	3.544 (3)	C21···H12A^i^	3.1255
Cl1···H18	2.7805	C21···H20^xiii^	3.4292
Cl1···H20	2.8122	C21···H24C^iv^	3.2186
O1···H2	1.7810	C21···H25C^iv^	3.3161
O1···H4A	2.7306	C22···H12B^xii^	3.4859
O1···H4B	2.4584	C22···H22A^xii^	3.2667
O1···H15	2.704 (19)	C22···H23C^xii^	3.5905
O2···H10A	2.4728	C22···H24A^vi^	3.5355
O2···H10B	2.6956	C22···H24B^vi^	3.2308
O2···H14	3.076 (16)	C23···H1^vii^	3.1526
O3···H1	2.4318	C23···H6B^iv^	3.3975
O3···H4	1.8652	C23···H22A^xii^	3.5874
O3···H12A	2.7362	C24···H4B^iii^	3.5806
O3···H12B	2.4712	C24···H22B^i^	3.3070
O4···H1	2.4024	C24···H22C^i^	3.4528
O4···H6A	2.4738	C25···H18^x^	3.2287
O4···H6B	2.6942	H1···O4^vii^	3.1608
C1···H2	2.6080	H1···C23^v^	3.1526
C1···H4	2.4692	H1···H6A^vii^	3.1070
C1···H15	2.75 (3)	H1···H23A^v^	2.6890
C2···H2	2.9270	H1···H23B^v^	3.2230
C2···H4A	3.0110	H1···H23C^v^	3.0387
C2···H4B	3.2466	H2···C18^iii^	3.3192
C2···H4	2.3780	H2···H10B^ii^	3.4272
C2···H6A	3.2378	H2···H17^iii^	3.3905
C2···H6B	3.0428	H2···H18^iii^	2.4809
C2···H14	3.411 (19)	H2···H25A^iv^	3.4155
C2···H15	2.64 (3)	H2···H25C^iv^	3.4842
C2···H24B	2.7872	H4A···H10A^ii^	3.0071
C3···H1	3.2857	H4A···H10B^ii^	3.2823
C3···H2	2.6198	H4B···C24^iii^	3.5806
C3···H6B	3.2414	H4B···H10B^ii^	3.3716
C3···H15	2.59 (3)	H4B···H22B^ii^	3.2691
C3···H24A	3.4462	H4B···H22C^ii^	3.3845
C3···H24B	2.8090	H4B···H24A^iii^	2.6583
C4···H6A	3.2847	H4···C14^v^	3.5679
C4···H6B	2.7049	H4···H6A^vii^	3.3756
C4···H15	3.51 (2)	H4···H14^v^	2.7543
C4···H24A	2.7083	H4···H21^v^	3.1385
C4···H24B	2.7094	H4···H23A^v^	2.8328
C4···H24C	3.3471	H6A···O3^v^	2.6688
C4···H25A	2.6831	H6A···C13^v^	3.3361
C4···H25B	2.6826	H6A···H1^v^	3.1070
C4···H25C	3.3297	H6A···H4^v^	3.3756
C6···H4A	2.6888	H6A···H14^ix^	3.5791
C6···H4B	3.2874	H6A···H23A^ix^	3.3147
C6···H4	3.0338	H6A···H23B^ix^	3.3524
C6···H24A	3.3360	H6B···Cl1^vi^	3.0186
C6···H24B	2.6727	H6B···C23^ix^	3.3975
C6···H24C	2.7078	H6B···H10A^ix^	3.0101
C6···H25A	2.6697	H6B···H23A^ix^	3.2557
C6···H25B	3.3338	H6B···H23B^ix^	2.7143
C6···H25C	2.7091	H10A···H4A^ii^	3.0071
C7···H1	2.4829	H10A···H6B^iv^	3.0101
C7···H4A	3.1881	H10A···H25A^iv^	3.2595
C7···H24B	2.8038	H10B···O1^ii^	2.6745
C7···H24C	3.4905	H10B···C3^ii^	3.4852
C8···H2	2.3998	H10B···C4^ii^	3.5823
C8···H4	2.9370	H10B···C17^vi^	3.3620
C8···H10A	3.2378	H10B···C18^vi^	3.3305
C8···H10B	3.0358	H10B···H2^ii^	3.4272
C8···H12A	3.0152	H10B···H4A^ii^	3.2823
C8···H12B	3.2493	H10B···H4B^ii^	3.3716
C8···H14	2.67 (3)	H10B···H17^vi^	3.1744
C8···H23A	2.8629	H10B···H18^vi^	3.1355
C9···H1	3.2747	H12A···C16^vi^	3.0750
C9···H12A	3.2242	H12A···C17^vi^	2.9514
C9···H14	3.04 (2)	H12A···C18^vi^	2.9391
C9···H23A	2.8454	H12A···C19^vi^	3.0146
C9···H23B	3.5311	H12A···C20^vi^	3.1142
C10···H2	3.0054	H12A···C21^vi^	3.1255
C10···H12A	2.7350	H12A···H17^vi^	3.4273
C10···H12B	3.3071	H12A···H18^vi^	3.4190
C10···H22A	3.3271	H12A···H20^v^	3.4853
C10···H22B	2.6797	H12B···C22^xii^	3.4859
C10···H22C	2.6633	H12B···H20^v^	3.0569
C10···H23A	2.6830	H12B···H22A^xii^	2.6641
C10···H23B	2.7203	H12B···H22B^xii^	3.5275
C10···H23C	3.3442	H12B···H24B^vii^	3.2747
C12···H10A	3.3044	H12B···H24C^vii^	3.0340
C12···H10B	2.7462	H14···O3^vii^	3.395 (18)
C12···H22A	2.7028	H14···O4^vii^	2.74 (3)
C12···H22B	3.3414	H14···H4^vii^	2.7543
C12···H22C	2.6861	H14···H6A^iv^	3.5791
C12···H23A	2.7048	H14···H25C^iv^	2.9474
C12···H23B	3.3398	H15···H22C^i^	2.8245
C12···H23C	2.6821	H15···H23C^v^	3.3563
C13···H1	2.5025	H17···O1^iii^	2.6842
C13···H4	2.6894	H17···H2^iii^	3.3905
C13···H10B	3.2593	H17···H10B^i^	3.1744
C13···H23A	2.8373	H17···H12A^i^	3.4273
C13···H23C	3.4566	H17···H17^iii^	3.1033
C14···H2	2.7098	H17···H22C^i^	2.8924
C14···H21	2.8306	H18···O1^iii^	3.0529
C15···H1	2.9862	H18···O2^iii^	2.5113
C15···H2	3.2616	H18···C25^viii^	3.2287
C15···H17	2.6127	H18···H2^iii^	2.4809
C15···H21	2.6806	H18···H10B^i^	3.1355
C15···H24B	3.3767	H18···H12A^i^	3.4190
C16···H14	2.65 (3)	H18···H25A^viii^	2.7464
C16···H18	3.2524	H18···H25B^viii^	3.0890
C16···H20	3.2591	H18···H25C^viii^	3.3419
C17···H15	2.66 (3)	H20···O3^vii^	3.5562
C17···H21	3.2194	H20···C20^xiii^	3.4842
C18···H20	3.2304	H20···C21^xiii^	3.4292
C19···H17	3.2119	H20···H12A^vii^	3.4853
C19···H21	3.2230	H20···H12B^vii^	3.0569
C20···H18	3.2300	H20···H20^xiii^	3.2364
C21···H14	2.78 (3)	H20···H21^xiii^	3.1193
C21···H15	3.36 (2)	H20···H22B^xi^	3.2572
C21···H17	3.2185	H20···H24C^iv^	2.9433
C22···H10A	2.7502	H21···O3^vii^	2.6046
C22···H10B	2.5576	H21···O4^vii^	3.5252
C22···H12A	2.5897	H21···H4^vii^	3.1385
C22···H12B	2.7693	H21···H20^xiii^	3.1193
C22···H23A	3.3326	H21···H24C^iv^	3.2766
C22···H23B	2.6584	H21···H25C^iv^	3.4292
C22···H23C	2.6978	H22A···C12^xii^	3.4517
C23···H10A	2.6139	H22A···C22^xii^	3.2667
C23···H10B	3.3344	H22A···C23^xii^	3.5874
C23···H12A	3.3328	H22A···H12B^xii^	2.6641
C23···H12B	2.6147	H22A···H22A^xii^	2.3851
C23···H22A	2.6798	H22A···H23C^xii^	2.8117
C23···H22B	2.6786	H22A···H24B^vi^	3.1369
C23···H22C	3.3321	H22A···H24C^vi^	3.5488
C24···H4A	3.3461	H22B···Cl1^xiv^	3.2133
C24···H4B	2.6497	H22B···C24^vi^	3.3070
C24···H6A	2.6115	H22B···H4B^ii^	3.2691
C24···H6B	3.3310	H22B···H12B^xii^	3.5275
C24···H15	3.30 (2)	H22B···H20^xiv^	3.2572
C24···H25A	3.3237	H22B···H24A^vi^	3.0483
C24···H25B	2.6787	H22B···H24B^vi^	3.1185
C24···H25C	2.6497	H22B···H24C^vi^	3.1990
C25···H4A	2.6001	H22C···C15^vi^	3.3353
C25···H4B	2.7312	H22C···C16^vi^	3.2586
C25···H6A	2.7533	H22C···C17^vi^	3.0627
C25···H6B	2.5914	H22C···C24^vi^	3.4528
C25···H24A	2.6767	H22C···H4B^ii^	3.3845
C25···H24B	3.3219	H22C···H15^vi^	2.8245
C25···H24C	2.6561	H22C···H17^vi^	2.8924
H1···H2	3.5717	H22C···H23C^xii^	3.5301
H1···H4	1.9467	H22C···H24A^vi^	3.2653
H1···H14	2.4037	H22C···H24B^vi^	2.8963
H1···H15	3.2263	H23A···O4^vii^	2.7257
H2···H10A	3.2527	H23A···C1^vii^	3.5318
H2···H10B	3.2362	H23A···C7^vii^	3.2924
H2···H14	3.0890	H23A···H1^vii^	2.6890
H2···H15	3.4192	H23A···H4^vii^	2.8328
H4A···H6A	3.5860	H23A···H6A^iv^	3.3147
H4A···H6B	2.5897	H23A···H6B^iv^	3.2557
H4A···H24A	3.5430	H23B···Cl1^xiv^	3.0987
H4A···H25A	2.4219	H23B···O4^iv^	3.5219
H4A···H25B	2.8373	H23B···C6^iv^	3.4066
H4A···H25C	3.4925	H23B···H1^vii^	3.2230
H4B···H6B	3.5940	H23B···H6A^iv^	3.3524
H4B···H15	3.5270	H23B···H6B^iv^	2.7143
H4B···H24A	2.4764	H23C···C1^vii^	3.5995
H4B···H24B	2.9061	H23C···C14^vii^	3.4805
H4B···H24C	3.5322	H23C···C15^vii^	3.3798
H4B···H25A	3.0352	H23C···C22^xii^	3.5905
H4B···H25B	2.5591	H23C···H1^vii^	3.0387
H4B···H25C	3.5902	H23C···H15^vii^	3.3563
H4···H6A	3.2689	H23C···H22A^xii^	2.8117
H4···H6B	3.3024	H23C···H22C^xii^	3.5301
H6A···H24A	3.5087	H23C···H24B^vii^	3.1984
H6A···H24B	2.8271	H24A···C4^iii^	3.5584
H6A···H24C	2.4542	H24A···C22^i^	3.5355
H6A···H25A	3.0355	H24A···H4B^iii^	2.6583
H6A···H25C	2.6056	H24A···H22B^i^	3.0483
H6B···H24B	3.5883	H24A···H22C^i^	3.2653
H6B···H24C	3.5251	H24A···H24A^iii^	3.0705
H6B···H25A	2.3928	H24A···H25B^iii^	2.9152
H6B···H25B	3.4781	H24B···C22^i^	3.2308
H6B···H25C	2.8506	H24B···H12B^v^	3.2747
H10A···H22B	2.5856	H24B···H22A^i^	3.1369
H10A···H22C	3.0503	H24B···H22B^i^	3.1185
H10A···H23A	2.8210	H24B···H22C^i^	2.8963
H10A···H23B	2.4624	H24B···H23C^v^	3.1984
H10A···H23C	3.5133	H24C···O3^v^	3.2782
H10B···H12A	2.6583	H24C···C20^ix^	3.0203
H10B···H22A	3.4558	H24C···C21^ix^	3.2186
H10B···H22B	2.7868	H24C···H12B^v^	3.0340
H10B···H22C	2.3661	H24C···H20^ix^	2.9433
H10B···H23A	3.5987	H24C···H21^ix^	3.2766
H10B···H23B	3.5251	H24C···H22A^i^	3.5488
H12A···H22A	2.8250	H24C···H22B^i^	3.1990
H12A···H22B	3.4839	H25A···Cl1^x^	3.3630
H12A···H22C	2.4046	H25A···O2^ix^	2.8705
H12A···H23C	3.5052	H25A···C18^x^	3.2196
H12B···H22A	2.6114	H25A···C19^x^	3.5403
H12B···H22C	3.0698	H25A···H2^ix^	3.4155
H12B···H23A	2.8708	H25A···H10A^ix^	3.2595
H12B···H23B	3.5008	H25A···H18^x^	2.7464
H12B···H23C	2.4241	H25B···C18^x^	3.1455
H14···H15	2.85 (4)	H25B···C19^x^	3.3584
H14···H21	2.3527	H25B···H18^x^	3.0890
H14···H23A	3.3554	H25B···H24A^iii^	2.9152
H15···H17	2.4558	H25B···H25B^iii^	2.9735
H15···H24A	3.4262	H25C···O2^ix^	3.1597
H15···H24B	2.4753	H25C···C14^ix^	3.3828
H17···H18	2.3063	H25C···C15^ix^	3.5282
H20···H21	2.3224	H25C···C16^ix^	3.2630
H22A···H23A	3.5645	H25C···C21^ix^	3.3161
H22A···H23B	2.9224	H25C···H2^ix^	3.4842
H22A···H23C	2.5220	H25C···H14^ix^	2.9474
H22B···H23A	3.5451	H25C···H18^x^	3.3419
H22B···H23B	2.4785	H25C···H21^ix^	3.4292
			
C2—C1—C8	115.54 (12)	C3—C4—H4B	108.669
C2—C1—C14	115.38 (17)	C5—C4—H4A	108.673
C8—C1—C14	112.14 (14)	C5—C4—H4B	108.662
C1—C2—C3	123.14 (14)	H4A—C4—H4B	107.607
C1—C2—C7	119.37 (14)	C5—C6—H6A	108.612
C3—C2—C7	117.35 (14)	C5—C6—H6B	108.599
O1—C3—C2	123.43 (15)	C7—C6—H6A	108.615
O1—C3—C4	115.18 (15)	C7—C6—H6B	108.621
C2—C3—C4	121.39 (15)	H6A—C6—H6B	107.574
C3—C4—C5	114.34 (14)	C9—C10—H10A	108.598
C4—C5—C6	106.51 (18)	C9—C10—H10B	108.584
C4—C5—C24	111.06 (18)	C11—C10—H10A	108.574
C4—C5—C25	110.12 (14)	C11—C10—H10B	108.582
C6—C5—C24	110.00 (16)	H10A—C10—H10B	107.551
C6—C5—C25	110.41 (19)	C11—C12—H12A	108.724
C24—C5—C25	108.7 (2)	C11—C12—H12B	108.715
C5—C6—C7	114.60 (16)	C13—C12—H12A	108.717
O4—C7—C2	122.66 (16)	C13—C12—H12B	108.714
O4—C7—C6	115.45 (16)	H12A—C12—H12B	107.633
C2—C7—C6	121.89 (17)	C1—C14—H14	114.8 (17)
C1—C8—C9	122.53 (17)	C15—C14—H14	117.5 (16)
C1—C8—C13	119.43 (13)	C14—C15—H15	118.8 (16)
C9—C8—C13	118.03 (17)	C16—C15—H15	115.4 (16)
O2—C9—C8	122.39 (17)	C16—C17—H17	119.307
O2—C9—C10	115.56 (14)	C18—C17—H17	119.318
C8—C9—C10	122.03 (18)	C17—C18—H18	120.298
C9—C10—C11	114.73 (13)	C19—C18—H18	120.306
C10—C11—C12	107.62 (16)	C19—C20—H20	120.532
C10—C11—C22	108.88 (14)	C21—C20—H20	120.546
C10—C11—C23	110.69 (18)	C16—C21—H21	119.403
C12—C11—C22	110.08 (18)	C20—C21—H21	119.393
C12—C11—C23	110.26 (13)	C11—C22—H22A	109.471
C22—C11—C23	109.28 (16)	C11—C22—H22B	109.480
C11—C12—C13	114.15 (19)	C11—C22—H22C	109.472
O3—C13—C8	122.53 (17)	H22A—C22—H22B	109.466
O3—C13—C12	115.58 (19)	H22A—C22—H22C	109.470
C8—C13—C12	121.88 (15)	H22B—C22—H22C	109.468
C1—C14—C15	127.28 (16)	C11—C23—H23A	109.477
C14—C15—C16	125.79 (16)	C11—C23—H23B	109.479
C15—C16—C17	119.23 (14)	C11—C23—H23C	109.464
C15—C16—C21	122.77 (19)	H23A—C23—H23B	109.479
C17—C16—C21	117.99 (18)	H23A—C23—H23C	109.466
C16—C17—C18	121.37 (16)	H23B—C23—H23C	109.462
C17—C18—C19	119.4 (3)	C5—C24—H24A	109.461
Cl1—C19—C18	118.95 (19)	C5—C24—H24B	109.474
Cl1—C19—C20	119.95 (13)	C5—C24—H24C	109.468
C18—C19—C20	121.10 (19)	H24A—C24—H24B	109.465
C19—C20—C21	118.92 (16)	H24A—C24—H24C	109.480
C16—C21—C20	121.2 (2)	H24B—C24—H24C	109.479
C9—O2—H2	109.471	C5—C25—H25A	109.468
C7—O4—H4	109.468	C5—C25—H25B	109.474
C2—C1—H1	103.982	C5—C25—H25C	109.469
C8—C1—H1	104.004	H25A—C25—H25B	109.471
C14—C1—H1	103.975	H25A—C25—H25C	109.476
C3—C4—H4A	108.685	H25B—C25—H25C	109.469
			
C2—C1—C8—C9	89.25 (18)	C1—C8—C13—O3	8.4 (2)
C2—C1—C8—C13	−89.82 (15)	C1—C8—C13—C12	−172.70 (11)
C8—C1—C2—C3	−80.3 (2)	C9—C8—C13—O3	−170.70 (12)
C8—C1—C2—C7	95.27 (15)	C9—C8—C13—C12	8.2 (2)
C2—C1—C14—C15	11.56 (18)	C13—C8—C9—O2	168.60 (13)
C14—C1—C2—C3	53.27 (18)	C13—C8—C9—C10	−10.0 (2)
C14—C1—C2—C7	−131.20 (14)	O2—C9—C10—C11	161.45 (13)
C8—C1—C14—C15	146.64 (12)	C8—C9—C10—C11	−19.9 (2)
C14—C1—C8—C9	−45.75 (17)	C9—C10—C11—C12	47.70 (18)
C14—C1—C8—C13	135.18 (13)	C9—C10—C11—C22	167.00 (13)
C1—C2—C3—O1	7.1 (3)	C9—C10—C11—C23	−72.86 (17)
C1—C2—C3—C4	−173.35 (14)	C10—C11—C12—C13	−49.17 (15)
C1—C2—C7—O4	−9.8 (3)	C22—C11—C12—C13	−167.69 (13)
C1—C2—C7—C6	171.04 (13)	C23—C11—C12—C13	71.7 (2)
C3—C2—C7—O4	165.96 (15)	C11—C12—C13—O3	−157.80 (12)
C3—C2—C7—C6	−13.2 (3)	C11—C12—C13—C8	23.23 (19)
C7—C2—C3—O1	−168.47 (15)	C1—C14—C15—C16	174.60 (11)
C7—C2—C3—C4	11.0 (3)	C14—C15—C16—C17	151.19 (14)
O1—C3—C4—C5	−157.25 (16)	C14—C15—C16—C21	−30.2 (2)
C2—C3—C4—C5	23.2 (3)	C15—C16—C17—C18	−179.88 (12)
C3—C4—C5—C6	−51.3 (3)	C15—C16—C21—C20	180.00 (13)
C3—C4—C5—C24	68.4 (3)	C17—C16—C21—C20	−1.4 (3)
C3—C4—C5—C25	−171.05 (17)	C21—C16—C17—C18	1.5 (2)
C4—C5—C6—C7	49.33 (17)	C16—C17—C18—C19	−0.8 (3)
C24—C5—C6—C7	−71.11 (17)	C17—C18—C19—Cl1	−179.51 (12)
C25—C5—C6—C7	168.87 (14)	C17—C18—C19—C20	−0.0 (3)
C5—C6—C7—O4	161.70 (13)	Cl1—C19—C20—C21	179.56 (10)
C5—C6—C7—C2	−19.1 (2)	C18—C19—C20—C21	0.1 (3)
C1—C8—C9—O2	−10.5 (2)	C19—C20—C21—C16	0.7 (3)
C1—C8—C9—C10	170.94 (11)		

Symmetry codes: (i) *x*, −*y*+1, *z*+1/2; (ii) −*x*+1, −*y*+1, −*z*+1; (iii) −*x*+1, *y*, −*z*+3/2; (iv) *x*, *y*+1, *z*; (v) −*x*+3/2, *y*−1/2, −*z*+3/2; (vi) *x*, −*y*+1, *z*−1/2; (vii) −*x*+3/2, *y*+1/2, −*z*+3/2; (viii) −*x*+1, *y*+1, −*z*+3/2; (ix) *x*, *y*−1, *z*; (x) −*x*+1, *y*−1, −*z*+3/2; (xi) *x*, −*y*+2, *z*+1/2; (xii) −*x*+3/2, −*y*+3/2, −*z*+1; (xiii) −*x*+3/2, −*y*+3/2, −*z*+2; (xiv) *x*, −*y*+2, *z*−1/2.

### Hydrogen-bond geometry (Å, º)

**Table:**

*D*—H···*A*	*D*—H	H···*A*	*D*···*A*	*D*—H···*A*
O2—H2···O1	0.82	1.78	2.596 (2)	173
O4—H4···O3	0.82	1.87	2.669 (2)	166
C18—H18···O2^iii^	0.93	2.51	3.418 (3)	165
C21—H21···O3^vii^	0.93	2.61	3.397 (3)	143

Symmetry codes: (iii) −*x*+1, *y*, −*z*+3/2; (vii) −*x*+3/2, *y*+1/2, −*z*+3/2.
